# Inhibition by female sex hormones of collagen degradation by corneal fibroblasts

**Published:** 2011-12-27

**Authors:** Hongyan Zhou, Kazuhiro Kimura, Tomoko Orita, Teruo Nishida, Koh-Hei Sonoda

**Affiliations:** 1 Department of Ophthalmology, Yamaguchi University Graduate School of Medicine, Ube City, Yamaguchi, Japan; 2 Department of Ophthalmology, China-Japan Union Hospital, Jilin University, Changchun City, China

## Abstract

**Purpose:**

Corneal fibroblasts contribute to collagen remodeling in the corneal stroma in part by mediating collagen degradation. Given that corneal structure is influenced by sex hormone status, we examined the effects of sex hormones on collagen degradation by corneal fibroblasts.

**Methods:**

Rabbit corneal fibroblasts were cultured in three-dimensional collagen gels with or without sex hormones including 17β-estradiol, progesterone, testosterone, and dehydroepiandrosterone (DHEA). Collagen degradation was determined by measurement of hydroxyproline after acid hydrolysis. The expression and activity of matrix metalloproteinases (MMPs) were evaluated by immunoblot analysis and gelatin zymography. The phosphorylation of mitogen-activated protein kinases (MAPKs) and the nuclear factor-kappa B (NF-κB) inhibitor NF kappa B Inhibitor-alpha (IκB-α) in corneal fibroblasts was examined by immunoblot analysis. Cell proliferation and viability were evaluated by measurement of bromodeoxyuridine incorporation and the release of lactate dehydrogenase, respectively.

**Results:**

17β-Estradiol and progesterone each inhibited interleukin **(**IL)–1β–induced collagen degradation by corneal fibroblasts in a concentration-dependent manner, whereas testosterone and DHEA had no such effect. MMP expression and activation in corneal fibroblasts exposed to IL-1β were also inhibited by 17β-estradiol and progesterone. These female sex hormones did not affect cell proliferation or viability. Both 17β-estradiol and progesterone inhibited the IL-1β–induced phosphorylation of p38 MAPK without affecting that of the MAPKs extracellular Signal-regulated Kinase (ERK) or c-jun N-terminal kinase (JNK). 17β-Estradiol also inhibited the IL-1β–induced phosphorylation of IκB-α.

**Conclusions:**

17β-Estradiol and progesterone inhibited MMP expression and activity in IL-1β–stimulated corneal fibroblasts and thereby suppressed collagen degradation by these cells.

## Introduction

Sex hormones belong to the family of steroid hormones, and their effects are mediated in large part through interaction with their specific nuclear receptors. Estrogen and progesterone are considered female sex hormones, whereas androgen and testosterone are considered male sex hormones. Although each of these hormones is present in both sexes, their levels differ between males and females. Sex hormones play multiple roles in the control of body homeostasis [1-3], including regulation of the expression of extracellular matrix proteins in arteries and the uterus [4,5]. Corneal structure, stiffness, and function have been found to differ between the sexes, with these differences being attributable in part to the effects of sex hormones [6,7]. Receptors for female and male sex hormones are present in the human cornea and likely contribute to the regulation of corneal structure and function [8,9].

Collagen architecture in the corneal stroma is an important determinant of corneal structure and function. Matrix metalloproteinases (MMPs) are zinc-dependent proteases that are responsible for the degradation of extracellular matrix proteins and which participate in various physiologic and pathological processes including development, tissue remodeling, wound healing, and cancer [10-13]. Collagen degradation by MMPs thus contributes to collagen remodeling associated with modulation of collagen architecture in tissues. Infiltration of inflammatory cells into the corneal stroma is promoted by the destruction of collagen fibrils in the stroma associated with corneal diseases [14,15]. Resident fibroblasts in the corneal stroma respond to various stimuli associated with injury, inflammation, and infection [16-18], and they contribute to the degradation of collagen fibrils through the release of MMPs. These enzymes have been shown to play a role in corneal inflammation and disease [19,20], and they are secreted by corneal fibroblasts in response to stimuli associated with such conditions [21,22].

The proinflammatory cytokine interleukin (IL)–1β is present in the tear fluid of individuals with corneal diseases such as bacterial keratitis and corneal ulcer as well as those with corneal chemical burns [23,24], and it contributes to the activation of corneal fibroblasts [25,26].

Corticosteroids also regulate various cellular functions and thereby maintain cell or tissue homeostasis. In particular, they modulate MMP expression and turnover of the extracellular matrix in several tissues [27-30]. We have previously shown that the synthetic corticosteroid dexamethasone inhibits IL-1β–induced collagen degradation by corneal fibroblasts as well as the IL-1β–induced expression and activation of MMPs in these cells [30,31]. Little is known of the effects of sex hormones on corneal remodeling by corneal fibroblasts, however. We have therefore now investigated the effects of sex hormones on collagen degradation and MMP expression by corneal fibroblasts.

## Methods

### Materials

Eagle’s minimum essential medium (MEM), Dulbecco’s phosphate-buffered saline (DPBS), dispase, antibiotic-antimycotic mixture, and trypsin-EDTA were obtained from Invitrogen-Gibco (Grand Island, NY); native porcine type 1 collagen (acid solubilized), 5× Dulbecco’s modified Eagle’s medium (DMEM), and collagen reconstitution buffer were from Nitta Gelatin (Osaka, Japan); fetal bovine serum (FBS) was from JRH Biosciences (Lenexa, KS); bovine plasminogen as well as collagenase, protease inhibitor cocktail, 17β-estradiol, progesterone, dehydroepiandrosterone (DHEA), and testosterone were from Sigma-Aldrich (St. Louis, MO); and recombinant human IL-1β was from R&D Systems (Minneapolis, MN). Mouse monoclonal antibodies to rabbit MMP-1 and MMP-3 were obtained from Daiichi Fine Chemicals (Toyama, Japan). Antibodies to phosphorylated NF kappa B Inhibitor-alpha (IκB-α), to p38 mitogen-activated protein kinase (MAPK), to phosphorylated p38 MAPK (Thr^180^, Tyr^182^), to c-Jun NH_2_-terminal kinase (JNK), to phosphorylated JNK (Thr^183^, Tyr^185^), to extracellular signal–regulated kinase 1 or 2 (ERK1/2), and to phosphorylated ERK1/2 (Thr^202^, Tyr^204^) were from Cell Signaling (Beverly, MA). Antibodies to IκB-α were obtained from Santa Cruz Biotechnology (Heidelberg, Germany). An enhanced chemiluminescence (ECL) kit as well as horseradish peroxidase–conjugated goat polyclonal antibodies to rabbit or mouse immunoglobulin G were from GE Healthcare (Piscataway, NJ). Culture plates (24- and 96-well) and 60-mm cell culture dishes were from Corning (Corning, NY); Coomassie brilliant blue and gelatin were from Bio-Rad (Hercules, CA); a cytotoxicity assay (CytoTox 96Non-Radioactive) was from Promega (Madison, WI); and a cell proliferation assay based on a colorimetric enzyme-linked immunosorbent assay for bromodeoxyuridine (BrdU) was from Roche (Basel, Switzerland). All media and reagents used for cell culture were endotoxin minimized.

### Cell isolation

Male Japanese albino rabbits (bodyweight, 2.0 to 2.5 kg) were obtained from Biotec (Saga, Japan). This study adhered to the ARVO Statement for the Use of Animals in Ophthalmic and Vision Research and was approved by the Animal Experimental Committee of Yamaguchi University School of Medicine. Rabbit corneal fibroblasts were isolated and maintained as described previously [32]. In brief, the enucleated eye was washed with DPBS containing antibiotic-antimycotic mixture, the endothelial layer of the excised cornea was removed mechanically, and the remaining corneal tissue was incubated with dispase (2 mg/ml, in MEM) for 1 h at 37 °C. After mechanical removal of the epithelial sheet, the remaining tissue was treated with collagenase (2 mg/ml, in MEM) at 37 °C until a single-cell suspension of corneal fibroblasts was obtained. The isolated corneal fibroblasts were cultured under a humidified atmosphere of 5% CO_2_ at 37 °C in 60-mm culture dishes containing MEM supplemented with 10% FBS. Proliferating cells were harvested for experiments at the subconfluent stage after four to seven passages in monolayer culture.

### Three-dimensional culture

Collagen gels were prepared as described [32]. In brief, corneal fibroblasts were harvested by exposure to trypsin-EDTA followed by centrifugation at 15,000× g for 5 min, and they were then suspended in serum-free MEM. Acid-solubilized collagen type I (3 mg/ml), 5× DMEM, collagen reconstitution buffer (0.05 M NaOH, 0.26 M Na_2_CO_3_, and 0.2 M HEPES [pH 7.3]), and corneal fibroblast suspension (2.2×10^6^ cells/ml in MEM) were mixed on ice at a volume ratio of 7:2:1:1. The resultant mixture (0.5 ml) was added to each well of a 24-well culture plate and allowed to solidify in an incubator containing 5% CO_2_ at 37 °C, after which 0.5 ml of serum-free MEM containing test reagents and plasminogen (60 μg/ml) was overlaid and the cultures were returned to the incubator for 36 h.

### Assay of collagenolytic activity

Collagen degradation was measured as previously described [26]. In brief, the supernatants from collagen gel incubations were collected, and native collagen fibrils with a molecular size of >100 kDa were removed by ultrafiltration. The filtrate was subjected to hydrolysis with 6 M HCl for 24 h at 110 °C, and the amount of hydroxyproline in the hydrolysate was determined by measurement of absorbance at 558 nm with a spectrophotometer.

### Immunoblot analysis

Immunoblot analysis of MMP-1 and MMP-3 was performed as previously described [32]. In brief, culture supernatants from collagen gel incubations performed in the presence of IL-1β and various concentrations of female sex hormones were subjected to SDS–PAGE on a 10% gel, and the separated proteins were transferred electrophoretically to a nitrocellulose membrane. Nonspecific sites of the membrane were blocked, and it was then incubated with antibodies to MMP-1 or to MMP-3. Immune complexes were detected with the use of horseradish peroxidase–conjugated secondary antibodies and ECL reagents. Immunoblot analysis of total or phosphorylated forms of ERK, p38 MAPK, JNK, or IκB-α was also performed as described previously [33]. In brief, cells (5×10^5^ per well of a 24-well plate) were cultured for 24 h in unsupplemented MEM and were then incubated first for 12 h with or without 17β-estradiol or progesterone and then for 30 min in the additional absence or presence of IL-1β (0.1 ng/ml). The cells were lysed in a solution containing 50 mM Tris-HCl (pH 7.5), 150 mM NaCl, 1 mM EDTA, 5 mM NaF, 1% Nonidet P-40, 0.5% sodium deoxycholate, 0.1% SDS, 1 mM Na_3_VO_4_, and 1% protease inhibitor cocktail, and the cell lysates (10 µg of protein) were then subjected to immunoblot analysis.

### Gelatin zymography

Gelatin zymography was performed as described previously [32]. In brief, culture supernatants (8 μl) from collagen gel incubations were mixed with 4 μl of nonreducing SDS sample buffer (125 mM Tris-HCl [pH 6.8], 20% glycerol, 2% SDS, 0.002% bromophenol blue), and 5 μl of the resulting mixture were subjected to SDS–PAGE in the dark at 4 °C on a 10% gel containing 0.1% gelatin. The gel was then washed with 2.5% Triton X-100 for 1 h before incubation for 18 h at 37 °C in a reaction mixture containing 50 mM Tris-HCl (pH 7.5), 5 mM CaCl_2_, and 1% Triton X-100. The gel was finally stained with Coomassie brilliant blue.

### Cell proliferation assay

Cells (2×10^4^ per well) seeded in a 96-well plate were incubated in MEM with or without sex hormones for 24 h, with BrdU added to the culture medium for the final 2 h. The medium was then removed, and the cells were processed for colorimetric detection of incorporated BrdU by measurement of absorbance at 370 nm with a microplate reader.

### Cytotoxicity assay

Cells (2×10^4^ per well) seeded in a 96-well plate were incubated in MEM with or without sex hormones for 24 h. The amount of lactate dehydrogenase (LDH) released into the culture medium was then measured with the use of an assay kit as described previously [34]. Absorbance at 490 nm was measured with a microplate reader.

### Statistical analysis

Data are presented as means±SEM and were analyzed with Dunnett’s multiple comparison test or Student’s unpaired *t* test. A p value of <0.05 was considered statistically significant.

## Results

### Inhibitory effects of female sex hormones on IL-1β–induced collagen degradation by corneal fibroblasts

**Figure 1:**
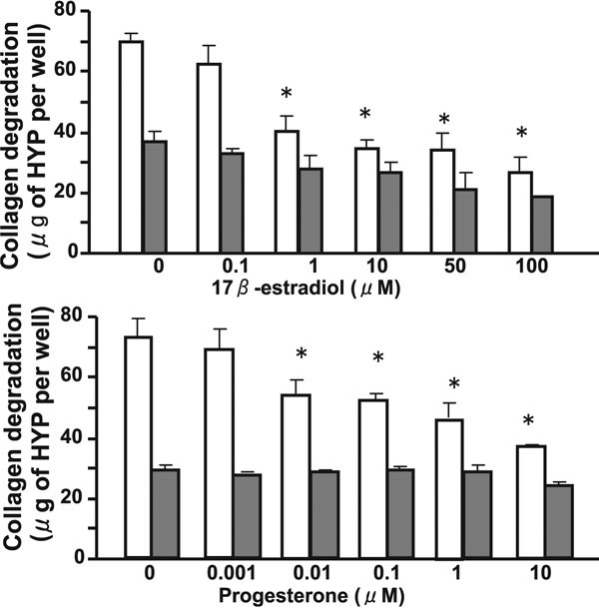
Concentration-dependent inhibition by 17β-estradiol and progesterone of IL-1β–induced collagen degradation by corneal fibroblasts. Cells were cultured in collagen gels with (white bars) or without (gray bars) IL-1β (0.1 ng/ml) and in the presence of the indicated concentrations of 17β-estradiol or progesterone for 36 h, after which the amount of degraded collagen was determined. Data are expressed as micrograms of hydroxyproline (HYP) per well and are means±SEM of quadruplicates from an experiment that was repeated three times with similar results. *p<0.05 (Dunnett’s test) versus the corresponding value for cells cultured without female sex hormones.

**Figure 2:**
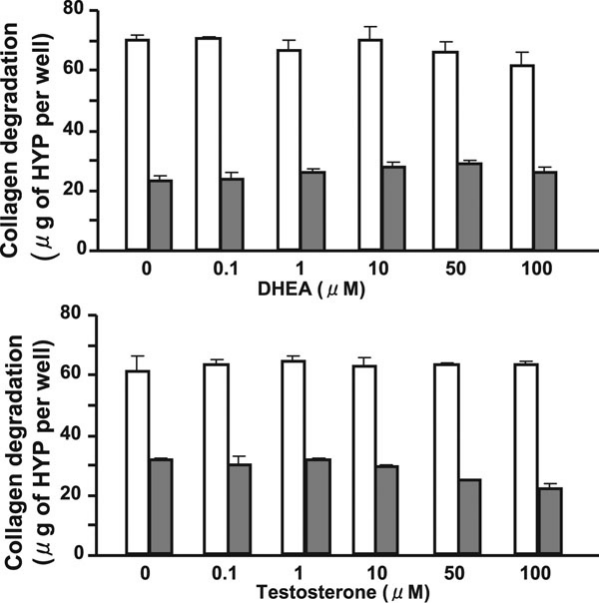
Lack of effect of DHEA or testosterone on IL-1β–induced collagen degradation by corneal fibroblasts. Cells were cultured in collagen gels with (white bars) or without (gray bars) IL-1β (0.1 ng/ml) and in the presence of the indicated concentrations of DHEA or testosterone for 36 h, after which the amount of degraded collagen was determined. Data are means±SEM of quadruplicates from an experiment that was repeated three times with similar results.

**Figure 3:**
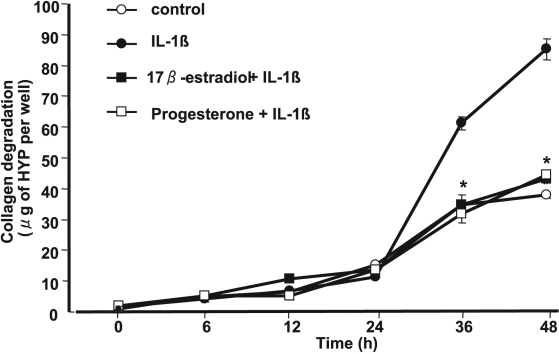
Time course of the inhibitory effects of 17β-estradiol and progesterone on IL-1β–induced collagen degradation by corneal fibroblasts. Cells were cultured for the indicated times in collagen gels in the absence or presence of IL-1β (0.1 ng/ml) and either 17β-estradiol (10 μM) or progesterone (10 μM) as indicated. The amount of degraded collagen was then determined. Data are means±SEM of quadruplicates from an experiment that was repeated three times with similar results. *p<0.05 (Student’s *t* test) for cells incubated with IL-1β plus 17β-estradiol or progesterone versus the corresponding value for those incubated with IL-1β alone.

We first examined the effects of sex hormones on IL-1β–induced collagen degradation by rabbit corneal fibroblasts in three-dimensional culture. The cells were incubated for 36 h with various concentrations of the sex hormones 17β-estradiol (0.1 to 100 μM), progesterone (0.001 to 10 μM), DHEA (0.1 to 100 μM), and testosterone (0.1 to 100 μM) in the absence or presence of IL-1β (0.1 ng/ml). Consistent with our previous observations [30], IL-1β increased the extent of collagen degradation by corneal fibroblasts. This effect of IL-1β was inhibited by the female sex hormones 17β-estradiol and progesterone in a concentration-dependent manner (Figure 1), whereas it remained unchanged in the presence of the male sex hormones DHEA or testosterone (Figure 2). The inhibitory effects of 17β-estradiol and progesterone on IL-1β–induced collagen degradation were significant at concentrations of ≥1 and ≥0.01 μM, respectively (Figure 1). Examination of the time course of IL-1β–induced collagen degradation in the absence or presence of 17β-estradiol (10 μM) or progesterone (10 μM) revealed that the inhibitory effects of these sex hormones were time dependent and were significant at 36 and 48 h (Figure 3).

### Inhibitory effects of 17β-estradiol and progesterone on the expression and activity of MMPs

**Figure 4:**
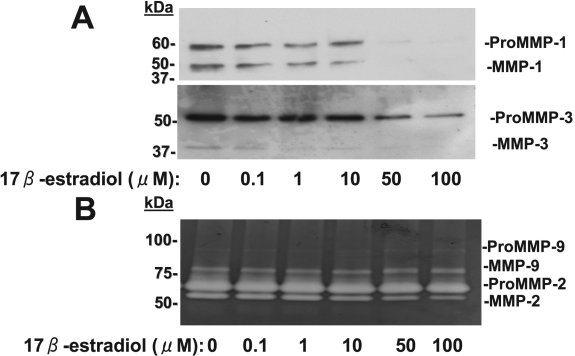
Effects of 17β-estradiol on MMP abundance and activity in culture supernatants of corneal fibroblasts stimulated with IL-1β. Cells were cultured in collagen gels in the presence of IL-1β (0.1 ng/ml) and the indicated concentrations of 17β-estradiol for 36 h, after which the culture supernatants were subjected to immunoblot analysis with antibodies to MMP-1 or to MMP-3 (**A**) or were analyzed by gelatin zymography (**B**). Bands corresponding to the pro and active forms of MMPs are indicated. Similar results were obtained in three separate experiments.

**Figure 5:**
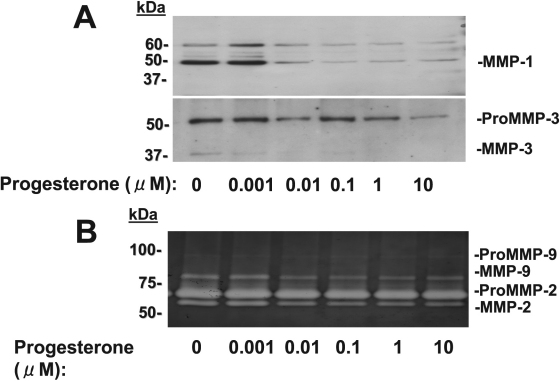
Effects of progesterone on MMP abundance and activity in culture supernatants of corneal fibroblasts stimulated with IL-1β. Cells were cultured in collagen gels in the presence of IL-1β (0.1 ng/ml) and the indicated concentrations of progesterone for 36 h, after which the culture supernatants were subjected to immunoblot analysis with antibodies to MMP-1 or to MMP-3 (**A**) or were analyzed by gelatin zymography (**B**). Similar results were obtained in three separate experiments.

We next examined the effects of 17β-estradiol and progesterone on MMP abundance and activity in culture supernatants of corneal fibroblasts by immunoblot analysis and gelatin zymography. The cells were cultured in collagen gels for 36 h with IL-1β (0.1 mg/ml) and in the presence of various concentrations of 17β-estradiol or progesterone. We previously showed that IL-1β increased the amounts of active or pro forms of MMP-1, MMP-2, MMP-3, and MMP-9 in the culture supernatants of corneal fibroblasts cultured in collagen gels [35]. Immunoblot analysis of culture supernatants revealed that 17β-estradiol (Figure 4A) and progesterone (Figure 5A) each reduced the abundance of both the active (49 kDa) and pro (61 kDa) forms of MMP-1 and both the active (45 kDa) and pro (57 kDa) forms of MMP-3 in a concentration-dependent manner. Gelatin zymography of the culture supernatants revealed that the amounts of the active forms of MMP-9 (77 kDa) and MMP-2 (57 kDa) were decreased by 17β-estradiol (Figure 4B) and progesterone (Figure 5B) in a concentration-dependent manner. These inhibitory effects of 17β-estradiol and progesterone appeared maximal at concentrations of ~50 and 0.01 μM, respectively.

### Effects of 17β-estradiol and progesterone on cell proliferation and viability

**Figure 6:**
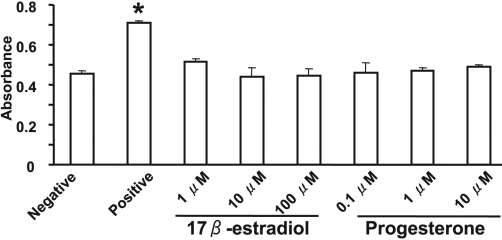
Lack of effect of 17β-estradiol or progesterone on the proliferation of corneal fibroblasts. Cells were cultured for 24 h in MEM and in the absence (negative control) or presence of various concentrations of 17β-estradiol or progesterone or of 10% FBS (positive control). Cell proliferation was evaluated by measurement of BrdU incorporation with an enzyme-linked immunosorbent assay. Data are means±SEM of quadruplicates from an experiment that was repeated three times with similar results. *p<0.05 versus negative control (Dunnett’s test).

**Figure 7:**
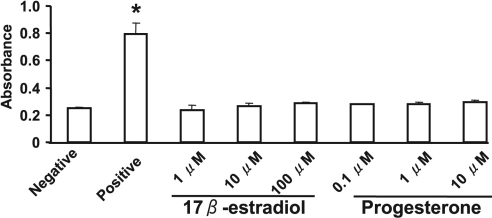
Lack of a cytotoxic effect of 17β-estradiol or progesterone on corneal fibroblasts. Cells were incubated for 24 h in MEM in the absence (negative control) or presence of various concentrations of 17β-estradiol or progesterone, after which the culture supernatants were assayed for LDH activity with a colorimetric assay and measurement of absorbance at 490 nm. The amount of LDH released from cells by a cell lysis solution was determined as a positive control. Data are means±SEM from three independent experiments. *p<0.05 versus negative control (Dunnett’s test).

We examined whether 17β-estradiol and progesterone might affect the proliferation of rabbit corneal fibroblasts. Incubation of the cells with 17β-estradiol at concentrations of 1 to 100 μM or with progesterone at concentrations of 0.1 to 10 μM for 24 h had no effect on cell proliferation (Figure 6). Similarly, measurement of LDH release revealed that neither 17β-estradiol nor progesterone had a cytotoxic effect on corneal fibroblasts (Figure 7).

### Effects of 17β-estradiol and progesterone on MAPK and NF-κB signaling pathways in corneal fibroblasts

**Figure 8:**
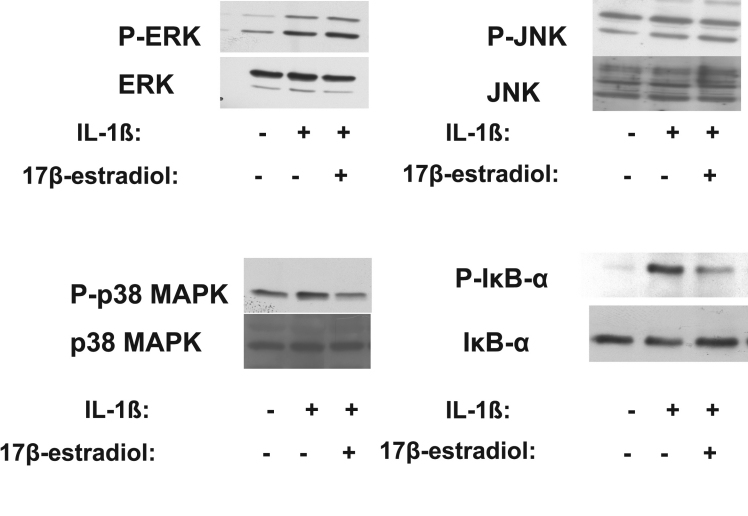
Effects of 17β-estradiol on IL-1β–induced MAPK and IκB-α phosphorylation in corneal fibroblasts. Cells were incubated in the absence or presence of 17β-estradiol (100 μM) for 12 h and then in the additional absence or presence of IL-1β (0.1 ng/ml) for 30 min. Cell lysates were then prepared and subjected to immunoblot analysis with antibodies to total or phosphorylated (P-) forms of ERK, JNK, p38 MAPK, or IκB-α. Data are representative of three independent experiments.

**Figure 9:**
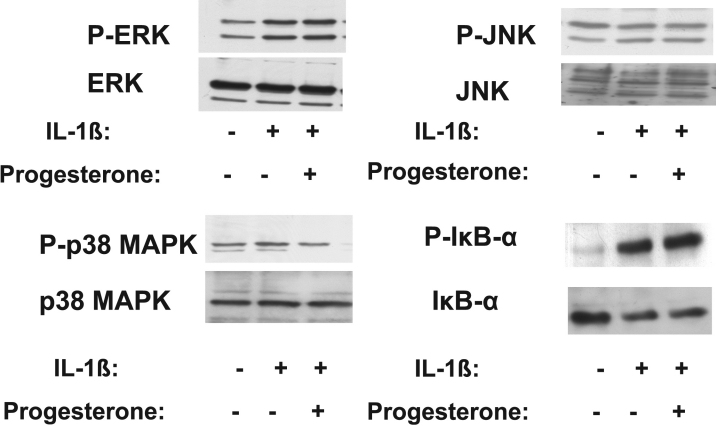
Effects of progesterone on IL-1β–induced MAPK and IκB-α phosphorylation in corneal fibroblasts. Cells were incubated in the absence or presence of progesterone (10 μM) for 12 h and then in the additional absence or presence of IL-1β (0.1 ng/ml) for 30 min. Cell lysates were then prepared and subjected to immunoblot analysis with antibodies to total or phosphorylated (P-) forms of ERK, JNK, p38 MAPK, or IκB-α. Data are representative of three independent experiments.

IL-1β activates MAPK and NF-κB signaling pathways in corneal fibroblasts [36]. We therefore investigated the effects of 17β-estradiol and progesterone on these signaling pathways in IL-1β–stimulated corneal fibroblasts. Cells were incubated in the absence or presence of 17β-estradiol (100 μM) or progesterone (10 μM) for 12 h and then in the additional presence of IL-1β (0.1 ng/ml) for 30 min. Immunoblot analysis showed that the phosphorylation level of each of the MAPKs ERK, JNK, and p38 as well as that of the NF-κB inhibitor IκB-α were increased by stimulation with IL-1β. The IL-1β–induced phosphorylation of p38 MAPK was inhibited by 17β-estradiol and progesterone, whereas that of IκB-α was inhibited by 17β-estradiol alone (Figure 8 and Figure 9). The phosphorylation of ERK and JNK induced by IL-1β was not affected by 17β-estradiol or progesterone.

## Discussion

We have here shown that 17β-estradiol and progesterone each inhibited IL-1β–induced collagen degradation by rabbit corneal fibroblasts in a concentration- and time-dependent manner, whereas testosterone and DHEA had no such effect. 17β-Estradiol and progesterone also suppressed the expression and activation of MMPs in corneal fibroblasts exposed to IL-1β in a concentration-dependent manner. These two female sex hormones did not affect the proliferation or viability of corneal fibroblasts. The IL-1β–induced phosphorylation of p38 MAPK in corneal fibroblasts was inhibited by 17β-estradiol and progesterone, whereas 17β-estradiol also inhibited the IL-1β–induced phosphorylation of IκB-α. These results thus suggest that female sex hormones inhibit collagen degradation by corneal fibroblasts through down-regulation of the expression and activity of MMPs. Furthermore, these effects may be mediated, at least in part, through inhibition of p38 MAPK and NF-κB signaling pathways.

The physiology of organs and tissues in the body differs between the sexes as a result of differences in sex hormones. These hormones regulate the extracellular matrix of various cell types [4]. Furthermore, menstruation affects visual acuity by inducing ocular changes in women [37]. The cornea of older men is also flatter than that of older women [38]. Estrogen, androgen, and progesterone receptors have been detected in corneal epithelial, stromal, and endothelial cells [8,9]. We have now shown that 17β-estradiol and progesterone inhibited IL-1β–induced collagen degradation by corneal fibroblasts. These female sex hormones also inhibited MMP expression and activation in these cells. In contrast, the male sex hormones testosterone and DHEA did not affect IL-1β–induced collagen degradation by corneal fibroblasts. These results suggest that female sex hormones may attenuate the degradation of stromal collagen under physiologic or pathological conditions. We previously showed that IL-1β promotes the expression and activation of MMP-1, −2, −3, and −9 in corneal fibroblasts with the use of immunoblot analysis and gelatin zymography [25,26,36]. With the same approaches, we now show that female sex hormones inhibit the IL-1β–induced expression and activation of these MMPs. Gelatin zymography is usually applied for the detection of gelatinases such as MMP-2 and MMP-9 [39]. In addition to MMP-2 and MMP-9, each of the enzymes MMP-1, MMP-8, and MMP-13 is also able to degrade gelatin. The signals for MMP-1, MMP-8, and MMP-13 in gelatin zymography, however, are expected to be fainter than those for MMP-2 and MMP-9 because gelatin is not their major substrate [40,41]. Ideally, though, gelatin zymography should be performed with proteinase standards.

We previously showed that dexamethasone inhibits MMP expression and activation in IL-1β–treated rabbit corneal fibroblasts [30]. Dexamethasone also inhibited the IL-1β–induced phosphorylation of ERK and JNK, but not that of p38 MAPK and IκB-α, in these cells [30,31,36]. We have now shown that 17β-estradiol and progesterone inhibited the IL-1β–induced phosphorylation of p38 MAPK, but not that of ERK or JNK, in rabbit corneal fibroblasts. 17β-Estradiol also inhibited the IL-1β–induced phosphorylation of IκB-α, whereas progesterone had no such effect. Sex hormones and glucocorticoids belong to the family of steroid hormones, but they exert different effects mediated by corresponding specific receptors [42,43]. Our results suggest that dexamethasone, 17β-estradiol, and progesterone interact with their specific receptors in corneal fibroblasts and thereby inhibit MMP expression and activation and consequent collagen degradation through differential regulation of intracellular signaling pathways.

Proinflammatory cytokines such as IL-1β and IL-6 contribute to corneal ulceration [44]. IL-1β promotes MMP expression and activation in corneal fibroblasts as well as consequent collagen degradation by these cells [25]. IL-1β is also present in the tear fluid of individuals with corneal diseases [23,45]. MMPs have been shown to play a role in corneal conditions such as bacterial keratitis, ocular surface inflammation, and injury [46-48]. Pterygium is more likely to occur in men than in women [49,50], and MMPs also contribute to the development and progression of this condition [51,52]. Our present demonstration that female sex hormones inhibit MMP expression and activation as well as collagen degradation in IL-1β–stimulated corneal fibroblasts suggests that these steroids may impede the progression of corneal inflammation and disease in women.

In summary, we have shown that the female sex hormones 17β-estradiol and progesterone each inhibit IL-1β–induced collagen degradation by corneal fibroblasts. These hormones also inhibited the expression or activation of MMP-1, MMP-2, MMP-3, and MMP-9, effects that may result from modulation of MAPK and NF-κB signaling pathways. Our results therefore suggest that female sex hormones warrant further investigation as potential drugs for the treatment of corneal diseases.
